# Books Reviewed

**Published:** 1879-05

**Authors:** 


### Books Reviewed.

*Clinical Diagnosis.* A Hand-book for Students and Practitioners of Medicine. Edited by JAMES FINLAYSON, M. D., with eighty-five illustrations. Philadelphia: Henry C. Lea, 1878.

We rarely recommend Hand-books, now so much in fashion. It seems to us that well-informed Physicians should be at least familiar with the Hand-book standard of Medicine, but in looking over the pages of this book, we are obliged to think many kind things of it, and to say that the student and physician will find chapters condensed, carefully written, comprehensive and complete, thus saving the time and labor required in the study of the more extensive works upon practical medicine.

The following is the list of contributors and their subjects, showing to our readers the standing of the Authors whose views are embodied in the work.

W. T. GAIRDNER, M. D., Professor of the Practice of Physic in the University of Glasgow: Physician to the Glasgow Western Infirmary, &c.

On the Physiognomy of Disease.

JAMES FINLAYSON, M. D., Physician and Lecturer on Clinical Medicine in the Glasgow Western Infirmary, &c.

On Case-Taking, Family History, &c.
On Symptoms of Disorder in the various Systems.
(Except in so far as specified below.)

WILLIAM STEPHENSON, M. D., Regius Professor of Midwifery in the University of Aberdeen.

On Disorders of the Female Organs.

ALEXANDER ROBERTSON, M. D., Physician and Superintendent of the Town's Hospital and City Parochial Asylum, Glasgow.

On Insanity.

SAMSON GEMMEL, M. B., Assistant to the Professor of the Practice of Physic in the University of Glasgow, and physician to the Dispensary of the Glasgow Western Infirmary.

On the Sphygmograph.

On the Physical Examination of the Chest and Abdomen.

JOSEPH COATS, M. D., Pathologist and Lecturer on Pathology in the Glasgow Western Infirmary, and also Physician to the Dispensary and its Throat Department.

On the Examination of the Fauces, Larynx, and Nares.

On the Method of Performing Post-Mortem Examinations.

---

*Lectures on Bright's Disease of the Kidneys, delivered at the School of Medicine of Paris.* By J. M. CHARCOT, Professor in the Faculty of Medicine of Paris; Physician to the Salpêtrière; Member of the Academy of Medicine; of the Clinical Society of London; of the Clinical Society of Buda-Pesth; of the Society of Natural Sciences, Brussels; President of the Anatomical Society; former Vice-President of the Society of Biology, &c., &c. Collected and published by Drs. BOURNEVILLE and SEVESTRE, editors of the Progrès Médical and translated, with the permission of the author, by HENRY B. MILLARD, M. D., A. M. New York: Wm. Wood & Co., 27 Great Jones St. 1878. H. H. Otis.

The author of this book has given the medical Profession a clear, concise and practical description of the pathology and histology of Bright's disease, not confounding it with any other disorder of the kidneys. He has also given colored illustrations of morbid conditions which augment its interest and value. He points out the clinical phenomena which aid in the recognition of amyloid degeneration of the kidney in the following words:

"It may be said that there are no symptoms which belong to it specifically. The phenomena associated with it are sometimes those of parenchymatous, and sometimes those of interstitial nephritis; sometimes, finally, the two classes of symptoms are found mingled together. Nothing, however, is easier, generally, than to establish the diagnosis, but this is founded almost exclusively upon extrinsic consideration.

"The existence of an habitual albuminuria and the persistence of a certain

œdema are sufficient in this case to enable us to recognize that we have to deal with a renal lesion, but only the presence of phenomena belonging to the amyloid diathesis reveals the special nature of the kidney affection. Thus, the patient is phthisical, suffers with prolonged suppuration or syphilitic cachexia. We recognize, besides, the existence of a considerable swelling of the liver and spleen; finally, the patient suffers from unmanageable diarrhœa, of a watery and painless character, proceeding from the amyloid affection of the arteries of the small intestines. When these conditions exist, it is easy to recognize and affirm the existence of amyloid degeneration of the kidney.

"For the rest, we have not up to the present time succeeded in recognizing distinctly, how in such a case, the parenchymatous or interstitial alterations of the kidney depend upon the amyloid alteration of the arteries, no more than we have in establishing the physiology of the symptoms which depend upon these alterations. These are questions the solution of which is reserved for the future."

The whole subject matter of the book is of too much importance and interest to be neglected by the ambitious, thoughtful physician, and no author can present the subject more concisely, scientifically and practically, though nothing is said of its treatment other than what is connected with its pathology and histology.

---

*A Manual for the Practice of Surgery.* By THOMAS BRYANT, F. R. C. S., Surgeon to and Lecturer on Surgery at Guy's Hospital Memb. Correspond. de la Société de Chirurgie de Paris, with six hundred and seventy-two illustrations. Second American from the *Third Revised and Enlarged English Edition.* Philadelphia: Henry C. Lea, 1879.

Our author makes the following quotations as a motto, and it is so apt and suggestive that we quote it entire, since its scope and spirit is so thoroughly carried into the text of the volume and so beautifully illustrated in its pages:

"Only the association of medicine with surgery forms the perfect physician. The physician who is deficient in the knowledge of one of these branches resembles a bird with but one wing." *Art of Life* (*Ayur Vede*), *early Sanscrit, first century of Christian era.*

"Surgery consists in curing disease rather than in the removal of it by mechanical means. But so differently do most think upon this subject that a surgeon who performs most operations and gives most pain is commonly thought the best."—*John Hunter, Ms. Lectures*, 1787.

Our author has very modestly called his work a "*Manual*" for the Practice of Surgery, but it is a comprehensive, condensed, beautifully illustrated work upon practical surgery of about 1,000 pages, adapted to the wants of the student, and at the same time a book of reference for the practical surgeon, of unsurpassed value, and every physician and surgeon will act wisely by adding it to his library. It has points of great interest too numerous for mention.

*Notes on the Treatment of Skin Diseases.*—By ROBERT LIVEING, A. M. and M. D. Cantab. F. R. C. P. Lond. Lately Physician and Lecturer to Middlesex Hospital and Physician in charge of the Skin Department. Fourth Edition, revised and enlarged. New York: Wm. Wood & Co., Publishers., 27 Great Jones St. 1878. H. H. Otis.

The author's preface gives the best description of this little work, and we copy it entire as showing its scope and object. It admirably meets the wants of many practitioners:

"These short notes on the Etiology and Treatment of Skin Diseases were prepared with a view to their private circulation amongst the students of my class in Cutaneous Medicine at the Middlesex Hospital. I have, however, published them at the request of some of my friends and former pupils. The Notes consist of a few general remarks on the Etiology, Diagnosis, Treatment, and Classification of Skin Diseases, followed by some short sketches on the nature, history, and best modes of dealing with ordinary affections. To this are added a Glossary of Terms in common use, and numerous Formulæ derived chiefly from the prescriptions of Hebra, Anderson, and the Pharmacopœia of the Skin Hospital, Blackfriars."

---

*A Manual of Physical Diagnosis.* By FRANCES DELAFIELD, M. D., and CHARLES F. STILLMAN, M. D. New York: Wm. Wood & Co.

This is truly a Manual of Physical Diagnosis, as the title implies. It would seem invaluable to the clinical teacher, and will be a great help to the student who is trying to obtain any distinct understanding of Physical Diagnosis. It is bound interleaved so that it can be taken into the wards of a hospital or into a clinic room and used as a note-book. The superimposed plates show the location and relations of internal organs, and of themselves alone make the manual *indispensable* to the students attending our various medical colleges. And I may be allowed to say that physicians of considerable experience will be benefited and entertained by the very beautiful superimposed plate illustrations of the subject. For a book of its kind we regard it unequalled.

———:o:———

